# *In vitro* investigation on lactic acid bacteria isolatedfrom Yak faeces for potential probiotics

**DOI:** 10.3389/fcimb.2022.984537

**Published:** 2022-09-16

**Authors:** Qingli Zhang, Meng Wang, Xin Ma, Zhijie Li, Chenghui Jiang, Yangyang Pan, Qiaoying Zeng

**Affiliations:** ^1^ College of Veterinary Medicine, Gansu Agricultural University, Lanzhou, Gansu, China; ^2^ Technology and Research Center of Gansu Province for Embryonic Engineering of Bovine and Sheep & Goat, Lanzhou, Gansu, China

**Keywords:** Yak, diarrhea, lactic acid bacteria, potential probiotics, antimicrobial activity, Caco-2

## Abstract

In order to evaluate the potential and safety of lactic acid bacteria (LAB) isolated from faeces samples of Ganan yak as probiotic for prevention and/or treatment of yak diarrhea, four strains of LAB including *Latilactobacillus curvatus* (FY1), Weissella cibaria (FY2), *Limosilactobacillus mucosae* (FY3), and *Lactiplantibacillus pentosus* (FY4) were isolated and identified in this study. Cell surface characteristics (hydrophobicity and cell aggregation), acid resistance and bile tolerance, compatibility, antibacterial activity and *in vitro* cell adhesion tests were also carried out to evaluate the probiotic potential of LAB. The results showed that the four isolates had certain acid tolerance, bile salt tolerance, hydrophobicity and cell aggregation, all of which contribute to the survival and colonization of LAB in the gastrointestinal tract. There is no compatibility between the four strains, so they can be combined into a mixed probiotic formula. Antimicrobial tests showed that the four strains were antagonistic to *Escherichia coli*, *Staphylococcus aureus*, and *Salmonella typhimurium*. Moreover, the *in vitro* safety of the four isolates were determined through hemolytic analysis, gelatinase activity, and antibacterial susceptibility experiments. The results suggest that all the four strains were considered as safe because they had no hemolytic activity, no gelatinase activity and were sensitive to most antibacterial agents. Moreover, the acute oral toxicity test of LAB had no adverse effect on body weight gain, food utilization and organ indices in Kunming mice. In conclusion, the four LAB isolated from yak feces have considerable potential to prevent and/or treat yak bacterial disease-related diarrhea.

## 1 Introduction

As is a common disease caused by bacterial invasion (*E. coli*, *salmonella species*, *S. aureus*, *Shigella species*, *Campylobacter species*), parasitic infections, dietary changes and viral infections, diarrhea is often associated with disturbance of the intestinal flora and damage to the intestinal mucosal barrier (Kim et al., 2021; Liu et al., 2022). Moreover, diarrhea is prevalent in calves, which may seriously affect the growth and health and even cause death of calves, resulting in high treatment and breeding costs in the breeding industry and hence considerable economic losses (Dioso et al., 2020; Liu et al., 2022). In modern livestock production, antibacterials are widely used for growth promotion and disease prevention or treatment (Kim et al., 2021). However, accumulating evidence indicates that the overuse and misuse of antimicrobial agents cause extensive food safety problems and environmental pollution, and resistance of antibacterials may selectively spread from animals to humans, posing a public health risk (Hu and Cheng, 2016). Due to the increasing antimicrobial resistance of pathogens, since 2006, the European Union (EU) ratified usage prohibition of antibacterials as animal growth supplements and disease prevention. At present, many countries are following suit (More, 2020). Therefore, there is an urgent need for non antibacterial substitutes to promote animal growth and prevent diseases.

The Food and Agriculture Organization (FAO) and the World Health Organization (WHO) defined probiotics as ‘live microorganisms that, when administered in adequate amounts, confer a health benefit on the host’ (Food and Agriculture Oraganization/ World Health Organization [FAO/WHO], 2006)). Probiotics can produce organic acids (mainly acetic acid and lactic acid), hydrogen peroxide, bacteriocins, bacteriocin-like inhibitory substances, short-chain fatty acids (SCFAs), conjugated linoleic acid (CLA), gamma-aminobutyric acid (GABA), vitamins (especially vitamin B and vitamin K) and other substances (Upadrasta and Madempudi, 2016; Zhang et al., 2022). Probiotics are a potential living biological therapy for maintaining gastrointestinal microecology by stimulating immunity, competing for nutrients, synthesizing antimicrobial peptides and metabolites for inhibiting epithelial and mucosal adhesion of pathogens, balancing unfavorable intestinal pH (Rolfe, 2000). Probiotics are considered as natural substitutes for antimicrobia. They are considered as capable of stabilizing the intestinal flora and normalizing peristaltic disorders. Besides, they could also inhibit the development of pathogenic microorganisms, prevent or reduce the course of bacterial, viral and antibiotic diarrhea, eliminate or reduce the symptoms of lactose intolerance and so on (Jarocki et al., 2020). Probiotics are widely used in treatment of human and animal gastrointestinal disorders because of their biological properties of promoting intestinal peristalsis and maintaining intestinal homeostasis (Wang et al., 2018; Jarocki et al., 2020; Zhang et al., 2020). The European Society for Pediatric Gastroenterology, Hepatology and Nutrition (ESPGHAN group) recommended the usage of probiotics as an adjunctive/preventive treatment for different types of diarrhea in pediatrics (Dioso et al., 2020). So far, probiotics are considered a safe and viable natural alternative to antibacterials in improving livestock performance due to their multiple beneficial effects on the host and have attracted a lot of attention from researchers (Li et al., 2019; Alayande et al., 2020).

Many projects have been undertaken to better understand the impact of probiotics on the intestinal ecosystem and its impact on health and disease. Probiotic feeding based on milk substitutes has the potential to control diseases, including neonatal calf diarrhea (Kayasaki et al., 2021). Liu et al. (2022) found that newborn calves in the complex probiotic group had tightly clustered intestinal bacterial communities and lower rates of diarrhea.

Diarrhea has been reported occurring in yak calves and is a major cause of calf death (Wang et al., 2018; Dong et al., 2020; Liu et al., 2022). Yak, which has a strong ability to adapt to the harsh natural environment, e.g. low temperature, food scarcity, especially low oxygen, is an ancient and primitive livestock species unique to the Qinghai Tibet Plateau. Not only used for farming and transportation, yaks can also local herdsmen with production and daily necessities such as milk, meat, wool, labor and fuel, which make them an important source of life and economy for herdsmen. The Gannan yak is one of the indigenous yak of China (Goshu et al., 2018).

However, the information we have about probiotic LAB from yak intestines is limited. Although some probiotics are generally recognized as safe (GRAS) there are a few reports of local or systemic infections, such as endocarditis and sepsis, that may be associated with the ingestion of certain lactobacilli (Bourdichon et al., 2012; Aroutcheva et al., 2016). Therefore, caution is still needed when choosing probiotics. To ensure the efficiency and safety of LAB for application, they must be systematically identified and characterized. Therefore, this study aims to providing a theoretical basis for the prevention and/or treatment of diarrhea associated with bacterial diseases in yaks *via* isolating and identifying LAB strains from Gannan yak, and evaluating their probiotic potential and safety.

## 2 Materials and methods

### 2.1 Sample collection, bacterial strains, cells and culture conditions

Samples were randomly collected from free-ranging yaks in Hezuo Forest Park in Gannan Tibetan Autonomous Prefecture. Twenty fecal samples were initially stored on-site by location in special sterile faecal sampling tubes filled with Phosphate buffer saline (PBS) (**Figure S1**). Then, samples were kept on ice and transported to Gansu Agricultural University in Lanzhou and stored at -80°C for further experiments.

*E. coli* (ATCC 25922), *S. aureus* (ATCC 25923), and *Salm. Typhimurium* (CMCC 50115) were purchased from Beijing Biobw Biotechnology Co., Ltd. They were incubated in Mueller Hinton (MH) agar plates or broth (Solarbio, China) and Luria Bertani (LB) broth (Solarbio, China) at 37°C aerobically for 24 h to study cell aggregation and antibacterial activity. The human colon cancer cell lines Caco-2 (BNCC350772) were purchased from the BeNa Culture Collection (BNCC; Beijing, China) and cultured in Dulbecco’s modified Eagle’s medium (DMEM with high glucose, Hyclone, Logan, UT, USA) supplemented with 10% fetal bovine serum (FBS, Pansera, Aidenbach, Germany) in 5% CO_2_ at 37°C.

### 2.2 Ethical statement and animal care

Experimental procedures adopted in this study were approved by the Animal Ethics Committee of College of Veterinary Medicine, Gansu Agricultural University (License no. GSAU-AEW-2019-0010).

Fifty (25 males and 25 females) specified pathogen-free (SPF) Kunming mice (Swiss albino mice origin, 4 weeks old, 18-22g) were obtained from Lanzhou Veterinary Research Institute, Chinese Academy of Agricultural Sciences. They were housed in standard plastic cages (5 per cage, segregated by sex) under controlled atmosphere (temperature 22 ± 3°C, humidity 55 ± 5%) with a light/dark cycle of 12/12 h. During the whole study, mice were freely consuming the same basic diet and plain water, and were monitored regularly for health status.

### 2.3 Isolation of LAB

The feces (ca. 1 g) were diluted by 10 times gradient in turn, and the bacterial suspensions of 10^−1^, 10^−2^ and 10^−3^ gradients were uniformly coated onto De Man, Rogosa, and Sharpe (MRS) agar (Solarbio, China) added with 2% (w/v) CaCO_3_ (Solarbio, China) incubated at 37°C anaerobically for 48 h. After the reaction of lactic acid with CaCO_3_, a clear area was formed around the colony. So, the milky white colony with clear zone was selected as tentative LAB for purification (Chang et al., 2007). All purified strains were mixed with equal volume of 50% (w/v) sterile glycerol and stored at -80°C for subsequent experiments.

### 2.4 Molecular identification

Molecular identification was performed according to Screening Criteria of Lactic Acid Bacteria for Feeding Aquatic Animals (TCSWSL016-2019). Four isolates were incubated overnight at 37 °C in MRS broth, and then genomic DNA was extracted using TIANamp bacteria DNA extraction kit (TIANGEN, Beijing, China). Molecular identification was performed by amplifying the 16S rRNA gene using universal primers (27F 5′-AGAGTTTGATCCTGGCTCAG -3′; 1492R, 5′-GGTTACCTTGTTACGACTT-3′) and previously described polymerase chain reaction (PCR) reaction conditions (Vaz-Moreira et al., 2009). PCR products were separated by 1.2% agarose gel electrophoresis and confirmed by sequencing (Genewiz, Suzhou, China). The obtained 16S rRNA sequences of the strains were compared with the EzBioCloud databases to identify the species (https://www.ezbiocloud.net/). Phylogenetic tree was constructed based on 16S rRNA sequences by MEGA software (version 7.0) with a Kimura two-parameter model for distance options and a Neighbor Joining (NJ) method for clustering with 1000 bootstrap replicates (Kumar et al., 2016).

### 2.5 Screening of probiotic strains

#### 2.5.1 Hydrophobicity

The degree of cell surface hydrophobicity of the LAB isolates was assessed *via* measuring microbial adhesion to hydrocarbons (MATH) in xylene (Macklin, Shanghai, China) according to the method in Zhang et al. (2022). Briefly, overnight cultures of four isolates were centrifuged at 5,000 × g for 3 min at 4 °C. The pellets were washed twice with PBS and resuspended in PBS, then the OD600 of the isolates was adjusted by spectrophotometry (Biochrom genequant, UK) in the range of 1.0 ± 0.1. Each bacterial suspension (3 mL) was mixed with 1 mL of xylene (Macklin, Shanghai, China), swirled for 2 min, then incubated at 37 °C for 1 h. The water layer was carefully aspirated and measured at OD600 (Biochrom genequant, UK). Cell surface hydrophobicity (%) was calculated the equation as follows:


$$\text{Hydrophobicity}\%=[(1-A_{t})/A_{0}]\times 100$$

Where A_0_ denotes the optical density at 0 h and A_t_ stands for the optical density at 1 h (Zeng et al., 2021).

The affinity of LAB to solvent was divided into hydrophilicity (< 10%), medium hydrophilicity (10-34%), medium hydrophobicity (35-70%) or high hydrophobicity (71-100%) (Qureshi et al., 2020)

#### 2.5.2 Cell aggregation

Aggregation properties of the selected LAB were performed according to the procedure described by El-Deeb et al. (2020) and Li et al. (2020). Briefly, four strains were incubated in MRS medium for 18 h at 37°C, then harvested by 5000 × g, centrifugation for 10 min. The pellets washed 3 times with PBS (pH 7.0), then resuspended in PBS and incubated (aerobic and static) at room temperature for 4 h. Then, the bacterial supernatant was pipetted out carefully for OD600 measurement. Three replicates were made for each strain. Autoaggregation (%) was calculated using the following equation:


$$\text{Autoaggregation}\%=[1-A_{0}/A_{t}]\times 100$$

Where A_0_ represents the absorbance at 0 h and A_t_ indicates the absorbance at 4 h.

Bacterial suspension for coaggregation was prepared as described above. Four isolates were assessed for their ability to co-aggregate with *E. coli*, *S. aureus* and *Salm. typhimurium*. The suspension of each LAB strain (2 mL) was mixed with the same volume of each indicator strain and three replicates were made. The OD600 of the suspensions were measured by spectrophotometry (Biochrom genequant, UK) at 0 h and 4 h. The coaggregation rates were calculated as follows.


$$\text{Coaggregation }\%=[(A_{p}+A_{i})-2A_{mix}(A_{p}+A_{i})]\times 100$$

Where A_p_ and A_i_ represent OD600nm of three standard bacterial strains and four isolates before mixing, A_mix_ is the pool absorbance at final time.

While A_p_ denotes the OD600 nm of the three indicator strains at 0 h, A_i_ is the OD600 nm of the four isolates before mixing, and A_mix_ is the absorbance after 4 h of the mixture.

#### 2.5.3 Acid resistance and bile tolerance

The viability of the isolates in acidic environments was determined according to the method described in Li et al. (2020) and Dowarah et al. (2018). Acid resistance was assessed with MRS broth (pH 3.0), and bile tolerance was tested with MRS broth supplemented with 0.3% (w/v) bile salt (Solarbio, China). Then, 2700 μl of each solution was mixed with 300 μl of each overnight culture in a 5 mL tube and incubated at 37°C for 4 h. The mixture was retrieved with a pipette and measured at OD600. Survival rates were calculated as follows:


$$\text{Survival rate}=A_{t}/A_{0}\times 100$$

Where A_0_ stands for the optical density at 0 h and A_t_ denotes the optical density at 4 h.

#### 2.5.4 Compatibility

The compatibility study was performed with minor modifications to previously described procedures (Aristimuno Ficoseco et al., 2018). Firstly, 10 mL of 1.5% sterilized agar was spread in each Petri dish, and after agar solidification, the Oxford cups were placed evenly on top. Then, 15 mL MRS solid medium was taken when it was cooled to 45°C and mixed vigorously with 200 μL of each LAB overnight culture and poured into Petri dishes. After cooling, the Oxford cups were gently removed and wells of 8 mm in diameter would appear on the agar. Then 50 µL of Cell-free culture supernatants (CFC) from each LAB strain was placed into each well, then the dishes were incubated aerobically at 37°C for 24 h. Finally, the diameter of the inhibition zone was observed and measured with a vernier caliper.

#### 2.5.5 Antibacterial activities

The agar well diffusion method was used to evaluate the antimicrobial activities of CFC from four isolates against *E. coli*, *S. aureus* and *Salm. typhimurium*. Four isolates were activated and cultured in MRS broth and indicator strains were incubated in LB broth at 37°C for 18 h. CFC of LAB were prepared by centrifugation (10000 × g, 10 min, 4 °C) and filtration (Millipore 0.22 μm). The bacterial suspension of indicator bacteria was adjusted to 10^7^ CFU/mL with LB and 100 µL of bacterial fluid was spread on the surface of MH agar plate. After the plate is dried, the Oxford cups (8 mm) were placed evenly on the plates and 100 µL CFC was loaded into the Oxford cup. MRS medium with pH 6.5 was added as control. Plates were incubated at 37°C for 24 h before measuring the inhibition zone. The inhibition zone diameter was scored as follows: negative (-), ≤9 mm; weak (+), 9-12mm; strong (++), 12-16mm; very strong (+++), ≥16mm (Qureshi et al., 2020).

#### 2.5.6 *In vitro* cell adhesion assay

The adhesion capacity was determined based on the previous method with minor adjustments (Li et al., 2020; Chen et al., 2022). Briefly, Caco-2 cells were seeded in 24-well plate (5×10^4^/well) and cultured in DMEM with 10% FBS to achieve a confluent monolayer cell. Four isolates were cultured in MRS broth for 24 h at 37°C and harvested using centrifugation (5000 × g, 3 min, 4°C). The pellets were washed twice in sterile PBS. Then, the bacterial solution was fluorescently labeled with carboxyfluorescein diacetate, succinimidyl ester (CFDA SE, Beyotime, China, 10 μg/mL) in PBS at 37°C for 10 min in the dark, and finally the cell density was adjusted to 10^8^ CFU/mL with DMEM medium. Cell monolayer were washed thrice with PBS, then 1 mL of DMEM serum-free medium and 50 µL of bacterial suspension were added into each well in three replicates, and 24-well plates were incubated for 1 h at 37°C in 5% CO_2_.

First, culture medium was aspirated and each well was carefully washed twice with sterile PBS to remove non-adherent bacteria. Then, 100 µL of trypsin was added into each well, the plates were incubated at 37°C for 10 min to completely digest the cells. Finally, cell culture medium was added into each well to stop the reaction. The fluorescence intensity was performed at 488 nm of excitation, and at 518 nm of emission wavelength by using the fluorescence microplate reader (SpectraMax i3x, Molecular Devices, USA). The percentage of adhesion was calculated as follows:


$$\text{Adhesion rate (}\%)=A_{a}/A_{b}\times 100$$

where A_b_ indicates the fluorescence intensity before adhesion and A_a_ represents the fluorescence intensity after adhesion.

### 2.6 Safety assessment

#### 2.6.1 Hemolytic activity

The four isolates and *S. aureus* were incubated in MRS medium and LB broth, respectively, for 24 h at 37°C. Each bacterial solution was streaked on blood agar plates containing 5% defibrinated sheep blood (Solarbio, China) and incubated at 37°C for 48 h. The clear zone around the *S. aureus* colony (β-hemolysis) was detected and used as a positive control (Moreno et al., 2018).

#### 2.6.2 Gelatinase activity

The gelatinase activity of LAB was conducted by using a previously reported method with minor adjustments (Perin et al., 2014; Rastogi et al., 2020). Briefly, 1 μL of 24 h incubated LAB was spotted on MRS agar with 5% (w/v) gelatin (Solarbio, China), and the plates were incubated anaerobically at 37°C for 72 h, then cooled at 4°C for 4 h. The opaque halo around the colony is considered to be a positive result of gelatinase production.

#### 2.6.3 Antimicrobial susceptibility

The disk diffusion method was used to determine the antimicrobial susceptibility patterns of all strains. Twelve antimicrobial paper disks (μg/disc) including ceftriaxone (30), ciprofloxacin (5), ampicillin (10), rifampicin (5), kanamycin (30), streptomycin (10), tetracycline (30), gentamicin (10), chloramphenicoll (30), erythromycin (15), clindamycin (2) and cephalothiophene (30) were purchased from Hangzhou Binhe Microorganism Reagent Co,. First, 100 µL of each bacterial solution (1×10^8^ CFU/mL) was evenly coated on MRS agar plates, and then antibacterial paper was placed on the plate at equal intervals. The plates were incubated at 37°C for 24 h. Finally, the diameter of the inhibition zone around the antibacterial paper was measured with vernier caliper and evaluated based on Clinical and Laboratory Standards Institute (CLSI, 2018). The sensitivity of LAB was classified as sensitive (S), intermediate (I), or resistant (R) to 12 antibiotics.

#### 2.6.4 Acute oral toxicity in mice

The acute oral toxicity test in mice was carried out following guidelines provided by Organisation of Economic Cooperation and Development (OECD) guidelines 423 (Organisation for Economic Cooperation and Development (OECD), 2001). After 5 days of acclimation, 50 mice were divided into 5 groups (5 males and 5 females). Four groups were gavaged with FY1, FY2, FY3 and FY4 at a concentration of 5×10^12^ CFU/mL, and the control group were fed with 200μL of saline. All mice were monitored for special attention during the first 4 hours and daily for 14 days thereafter for clinical signs or behavioral changes, body weight, food intake, and mortality. After mice were sacrificed, organs were taken to observe lesions, weighed and organ indices (heart, liver, spleen, lung, kidney, uterus, ovary or testis) were calculated for LAB safety assessment. Organ index were determined as follows: organ weight/mouse body weight.

### 2.7 Statistical analysis

Phylogenic tree were conducted using the MEGA 7 software and graphs were plotted by Originpro 2019b and GraphPad Prism 8.0. Date were expressed as mean ± standard deviation (SD) of at least three independent experiments. Statistical analysis was performed using SPSS 26.0, and statistical significance was defined as P<0.05 by one-way ANOVA following least significant difference (LSD) and Waller-Duncan’s post-treatment multiple comparisons.

## 3 Results

### 3.1 Isolation of LAB

**Figure 1:**
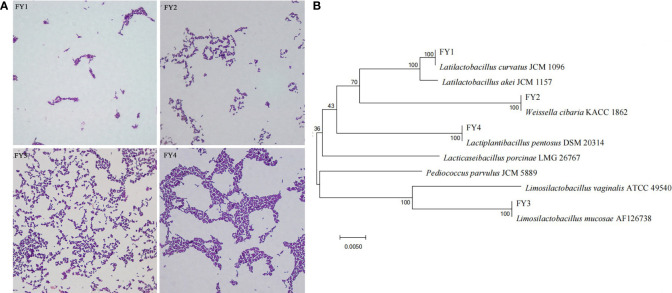
Phylogenetic tree of four isolates based on Neighbor-Joining distance analysis of 16S rRNA gene sequences. **(A)** The diagram shows the results of Gram staining for four isolates: FY1 (*L. curvatus*), FY2 (*W. cibaria*), FY3 (*L. mucosae*), and FY4 (*L. pentosus*). **(B)** Phylogenetic tree constructed by using a neighbor-joining method on the basis for 16S rRNA gene sequences.

After purification, four presumptive LAB (FY1, FY2, FY3, FY4) were selected according to the size of calcium dissolving ring and colony morphology. Gram-staining and microscopic examinations revealed that 4 strains were Gram-positive bacilli (**Figure S2**). Sequence analysis of 16S rRNA revealed that FY1, FY2, FY3, FY4 had 99-100% similarity with, *Latilactobacillus curvatus* (*L.* curvatus), *Weissella cibaria *(*W. cibaria), Lactiplantibacillus pentosus* (*L. pentosus*), and *Limosilactobacillus mucosae* (*L. mucosae*), respectively (**Figure S3**, **Data Sheet 1**). Phylogenetic trees (**Figure 1**) were constructed based on Neighbor-Joining algorithm and Kimura two-parameter model using the MEGA 7.0.

### 3.2 Accession numbers

The 16S rRNA nucleotide sequences of four strains were deposited in the GenBank database under the following accession numbers: FY1: *Latilactobacillus curvatus* (ON758920), FY2: *Weissella cibaria *(ON758921), FY3: *Lactiplantibacillus pentosus* (ON758922), and FY4: *Limosilactobacillus mucosae* (ON758923).

### 3.3 Hydrophobicity

**Figure 2:**
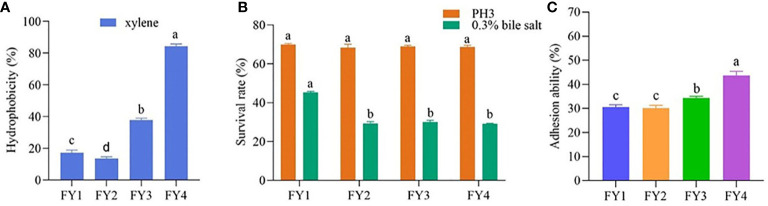
The hydrophobicity, acid and bile salt resistance and adhesion ability of LAB isolates. **(A)** Hydrophobicity percentages of LAB isolates to xylene **(B)** The acid and bile salt tolerance of LAB isolates. Values expressed as mean ± SD. Different letters represent significant difference, P< 0.05. **(C)** Percent adhesion values of LAB to Caco-2. Values expressed as mean ± SD. Different letters represent significant difference, P < 0.05.

**Figure 2A** shows that four isolates have varied cell surface hydrophobicity xylene. FY4 exhibited highly hydrophobic (84.33%), FY3 showed moderately hydrophobic (37.79%), while FY2 and FY1 had moderately hydrophilic(17.32%, 13.65%).

### 3.4 Acid resistance and bile tolerance

Acid resistance and bile tolerance of the four strains were shown in **Figure 2B**. Acid resistance of the four strains are very approximate, FY1 (69.96%), FY2 (68.29%), FY3 (68.86), and FY4 (68.59%). After 3 h exposure to bile salt condition, FY1 showed the highest bile tolerance (45.24%), followed by FY3 (30.10%), FY2 (29.33%), and FY4 (29.09%).

### 3.5 Cell aggregation

The percentage of autoaggregation and coaggregation of four isolates against three pathogenic bacteria were shown in **Table 1**. All the four strains have large ranges of autoaggregation ability (15.67-49.19%) and coaggregation ability (18.76-38.44%) to three pathogens. Among them, FY4 exhibited high autoaggregation ability (49.19%) and coaggregation ability with *E. coli* (38.43%), *S. aureus*(38.44%), and *Salm. Typhimurium* (34.81%).

**Table 1:** The percentage of autoaggregation and coaggregation with *E. coli*, *S. aureus*, and *Salm. typhimurium* by four isolates.

Isolates	Autoaggregation	Coaggregation		
		*E. coli*	*S. aureus*	*Salm. typhimurium*
FY1	15.67 ± 0.29 d	24.68 ± 2.65 c	23.00 ± 0.54 c	22.59 ± 0.38 b
FY2	23.88 ± 0.47 b	31.79 ± 0.38 b	24.92 ± 0.87 b	21.93 ± 0.72 b
FY3	19.24 ± 0.48 c	26.28 ± 0.99 c	26.13 ± 0.85 b	18.76 ± 0.34 c
FY4	49.19 ± 0.10 a	38.43 + 0.20 a	38.44 ± 0.41 a	34.81 ± 0.64 a

Values expressed as mean ± SD. Different letters represent significant difference, P < 0.05.

### 3.6 Compatibility

No inhibition halo was observed from the compatibility results among the four strains, indicating that four isolates can be combined as a mixed probiotic formula (**Figure S4**).

### 3.7 Antibacterial activities

The antibacterial activity results of four strains are shown in **Table 2**. FY3 exhibited the strongest antimicrobial activity towards *E. coli* (15.83 mm) and *S. aureus* (15.33 mm); FY4 showed the most potent antimicrobial activity to *Salm. typhimurium* (12.10 mm).

**Table 2:** The inhibition zone diameters (mm) of four strains against *E. coli*, *S. aureus *and *Salm. typhimurium.*

Isolates	Indicator pathogens					
	*E. coli*		*S. aureus*		*Salm. typhimurium*	
FY1	9.60 ± 0.16	+	10.43 ± 0.33	+	9.77 ± 0.21	+
FY2	10.77 ± 0.25	+	10.83 ± 0.35	+	9.87 ± 0.31	+
FY3	15.83 ± 0.29	++	15.33 ± 0.29	++	11.83 ± 0.35	+
FY4	12.83 ± 0.35	++	14.67 ± 0.42	++	12.10 ± 0.36	++

Values expressed as mean ± SD. Less than or equal to 9 mm (negative, -), 9-12mm (weak, +), 12-16mm (strong, ++), and more than 16mm (very strong, +++).

### 3.8 *In vitro* cell adhesion assay

The adhesion ability of four isolates to Caco-2 cells was shown in **Figure 2C**. Four isolates showed varying adhesion properties, ranging from 30.06% to 43.69%. FY4 has the maximum adhesion property (43.69%), followed by FY3 (34.33%), FY1 (30.61%) and FY2 (30.06%).

### 3.9 Safety assessment

#### 3.9.1 Antimicrobial susceptibility

**Figure 3:**
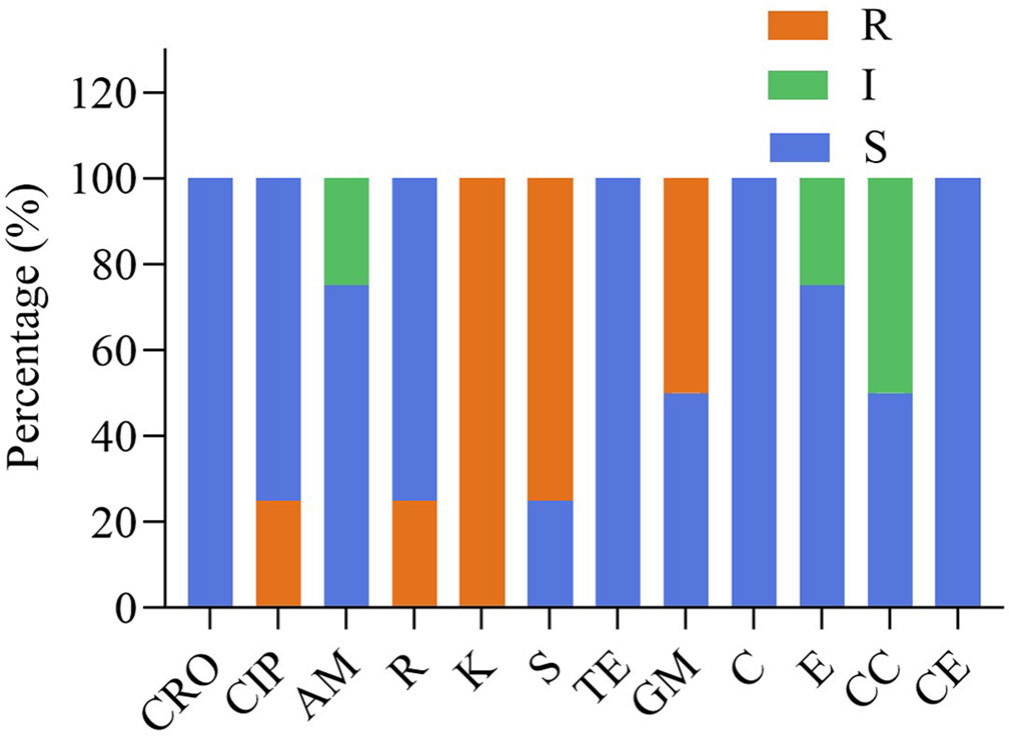
The antibiotic resistance of the LAB isolates against 12 tested antibiotics. Ceftriaxone (CRO), ciprofloxacin (CIP), ampicillin (AM), rifampicin (R), kanamycin (K), streptomycin (S), tetracycline (TE), gentamicin (GM), chloramphenicol (C), erythromycin (E), clindamycin(CC), cephalothiophene (CE). The total number of LAB strains was taken as 100%. S, sensitive; I, intermediately resistant; R, resistant.

The susceptibility of four isolates to 12 different antimicrobials are provided in **Table 3** and **Figure 3**. Four strains were susceptible to ceftriaxone, tetracycline, chloramphenicol and cephalothiophene, but showed resistant to kanamycin. FY1 showed intermediate susceptibility to erythromycin and clindamycin, resistant to ciprofloxacin, kanamycin, streptomycin and gentamicin. FY2 showed intermediate susceptibility to clindamycin, but resistant to kanamycin, streptomycin and gentamicin. FY3 showed intermediate susceptibility to ampicillin, but resistant to rifampicin, kanamycin, and streptomycin. FY4 was sensitive to all antimicrobials except kanamycin.

**Table 3:** Antibiotic susceptibility of selected LAB strains.

Strains	Antibiotic susceptibility zone of inhibition in mm													
		CRO	CIP	AM	R	K	S	TE	GM	C	E	CC	CE
FY1		S	R	S	S	R	R	S	R	S	I	I	S
FY2		S	S	S	S	R	R	S	R	S	S	I	S
FY3		S	S	I	R	R	R	S	S	S	S	S	S
FY4		S	S	S	S	R	S	S	S	S	S	S	S

Ceftriaxone (CRO), ciprofloxacin (CIP), ampicillin (AM), rifampicin (R), kanamycin (K), streptomycin (S), tetracycline (TE), gentamicin (GM), chloramphenicol (C), erythromycin (E), clindamycin (CC), cephalothiophene (CE). Erythromycin results based on R ≤ 13 mm; I: 13–23 mm; S ≥ 23 mm. Gentamycin results based on R ≤ 6 mm; I, 7–9 mm; S ≥ 10 mm. Streptomycin results based on R ≤ 11 mm; I, 12–14 mm; S ≥ 15 mm. S, susceptible (zone diameter, ≥21). I: intermediate (zone diameter, 15–20 mm); R, resistant (zone diameter, ≤15 mm).

### 3.10 Hemolytic activity

There was obvious hemolytic zone (β-hemolysis) around *S. aureus* colony on blood agar. While, colonies of four isolates had no zone effect (γ-hemolysis), indicating that they had no hemolytic activity (**Figure S5**).

### 3.11 Gelatinase activity

Four isolates did not have the property of breaking down gelatin, thus, they were considered as safe (**Figure S6**).

### 3.12 Acute toxicity in mice

**Figure 4:**
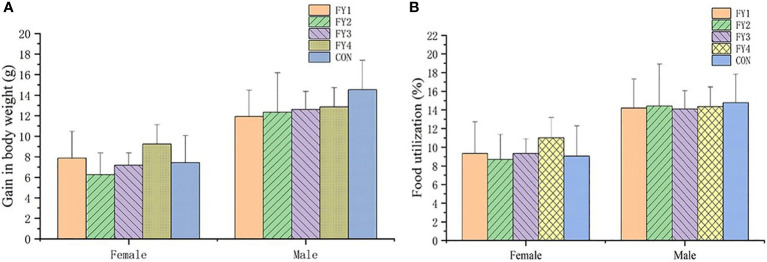
Effect of LAB on body weight gain and food utilization rate of experimental mice. **(A)** Body weight gain of female and male mice in each group. **(B)** Food utilization rate of female and male mice in each group.

**Figure 5:**
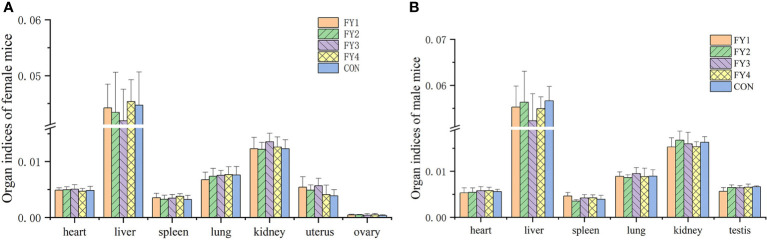
Organ indices of mice in each experimental group. **(A)** Organ indices of female mice (heart, liver, spleen, lung, kidney, uterus and ovary) **(B)** Organ indices of male mice (heart, liver, spleen, lung, kidney and testis).

No adverse effects or mortality were observed in the acute toxicity assay in mice. Compared with the control group, there was no significant difference in weight gain and food utilization rate of mice in the experimental group (**Figure 4**). In addition, no significant differences were found in the organ index of the same organ in each group (**Figure 5**).

## 4 Discussion

Our experimental designing were mainly based on previously published studies, Guidelines for the Evaluation of Probiotics in Food (Food and Agriculure Organization/ World Health Organization [FAO/WHO], 2006), Criteria TCSWSL016-2019 and OECD guidelines 423. The research can be divided into four sections: (1) identification of bacteria, (2) *In vitro* potential testing of probiotics, (3) proving *in vitro*ly safe probiotics, (4) proving *in vitro*ly safe probiotics. In our study, four LAB (*L. curvatus*, *W. cibaria*, *L. pentosus* and *L. mucosae*) were isolated, identified and evaluated for probiotic properties.

Hydrophobicity is an important property of probiotics that requires consideration during potential probiotic candidates selection (Reuben et al., 2019). Many studies have shown that hydrophobicity plays a crucial role in bacterial aggregation, colony formation, and initial cell adhesion to host cells (Myint et al., 2010; Pachla et al., 2021). In this study, only FY4 exhibited high hydrophobicity, which is consistent with previous studies that only a few LAB isolated from cattle showed high hydrophobicity (Otero et al., 2006; Maldonado et al., 2012). Hydrophobicity correlates with the physical and chemical properties of the bacteria, with high hydrophobicity indicating the presence of hydrophobic components on or embedded in the bacterial surface. When hydrophobicity exceeds 40%, probiotics are considered to have greater host adhesion and better pathogen competition inhibition (Lin et al., 2020). Studies have shown that hydrophobicity is a strain-specific characteristic and that hydrophobicity values differ significantly even between strains of the same LAB (Tokatli et al., 2015; Boranbayeva et al., 2020).

Probiotic strains need to tolerate acidic and bile salt environments in order to remain viable during gastrointestinal transport and maintain functional in the intestine (Yao et al., 2021). The pH of gastric acid fluctuates from 1.5 to 4.5, with a medium value of 3.0, and the range of bile salt concentration in the upper intestine is 0.03-0.3% (wt/vol) (Ding et al., 2017). Like many studies, we assayed the tolerance of isolates in pH 3 and in 0.3% bile salt. In this study, four isolates all had close viability under acidic condition, but different viability in bile salt, which may be attributed to the expression of resistance-associated proteins in strains and species.

Autoaggregation, coaggregation and hydrophobicity of bacteria are considered to be important properties affecting their colonization in the intestine (Diale et al., 2021). Autoaggregation is the ability of the same cell types to self-adhere, and coaggregation is the combination of organisms of different species (Del Re et al., 2000; Kolenbrander, 2000). A high self-aggregation capacity ensures that microorganisms achieve higher population density and stability in the host’s gut, reducing exposure to unfavorable conditions (Slizewska et al., 2021). A high coaggregation capacity can prevents the host’s gastrointestinal tract from being colonized by pathogens (Dlamini et al., 2019). Some studies reported significant correlations between self-aggregation and hydrophobicity in some Lactobacillus strains, while others pointed to positive correlations between cell hydrophobicity and the presence of protein surface coatings, leading to aggregation and adhesion capacity (Aleksandrzak-Piekarczyk et al., 2015; Cozzolino et al., 2020). In this study, FY4 exhibited adhesion ability, high hydrophobicity, strong autoaggregation and coaggregation ability. These results are consistent with previous studies that strong adhesion is correlated with high hydrophobicity and high aggregation ability (Wang et al., 2018; Sharma et al., 2021).Antibacterial activity against pathogens is another functional requirement for probiotics. The antibacterial effect of probiotics is complex and multifactorial, mainly by fighting against binding sites and nutrients, stimulating the host immune system to competitively reject pathogens and producing inhibitory metabolites against unfavorable microorganisms (Slizewska et al., 2021). Its metabolisms with antibacterial effect are mainly organic acids (mainly acetic acid and lactic acid), hydrogen peroxide, ethanol, bacteriocin and bacterioids (Azhar et al., 2017). Like many previous studies, the CFC (metabolites) of LAB for *in vitro* bacterial inhibition experiments were adopted in this study, and the results showed that four isolates have antibacterial activity against enteric pathogenic bacteria.

The adhesion of LAB in the intestine is not only beneficial for intestinal colonization and pathogens elimination, but also for immunomodulation and synthesis of healthy molecules (Donaldson et al., 2016; Slizewska et al., 2021). The adhesion of probiotics to the host is related to their cell surface properties, such as charges on bacterial cell and host cell, teichoic acids, extracellular polysaccharides, pili, and cell wall-anchored proteins (Donaldson et al., 2016; Slizewska et al., 2021).

*In vitro* adhesion of LAB with Caco-2 cells, which has been widely applied in the identification of potential probiotics (Yeo et al., 2016), was adopted in this study. The adhesion rate of four isolates was 30.06% to 43.69%. Consistent with the previous studies that cell adhesion is proportional to hydrophobicity, FY4 has the highest cell adhesion and hydrophobicity in this study Albeit LAB are GRAS microorganisms, and safety properties should be evaluated prior to administration. In this study, four isolates were tested on their capabilities for hemolytic analysis, gelatinase activity, and antibacterials resistance. Some bacteria are known to produce enzymes that break down phospholipids and cause rupture of the cell membrane of red blood cells (RBCs). Hemolytic activity is considered to be an important virulence factor in pathogenesis, facilitating the acquisition of iron or other “growth factors” to pathogens, which leads to host anemia or edema, etc. (Vesterlund et al., 2007). Therefore, bacterial hemolytic activity is the first *in vitro* safety parameter need to be evaluated. There are three types of bacterial hemolytic activities, α-hemolysis (green-hued zone around colonies), β-hemolysis (transparent zone around colonies), and γ-hemolysis (no change around colonies). Alpha hemolysis is not true hemolysis, since it actually is the oxidation of hemoglobin to green methemoglobin by the hydrogen peroxide produced by the bacteria, which gives the bacterial colonies a green color. Beta-hemolysis is the complete lysis of RBCs around the colony and in the medium below, giving the area a pale (yellow) and transparent appearance. Gamma-hemolysis is the absence of hemolytic activity and causes no change around the colony (Pradhan et al., 2020).

Gelatinase is a kind of Zn metalloproteinase secreted by pathogenic bacteria. It can effectively attack the host by digesting the protein components of tissue, so as to facilitate the spread of bacteria (Salamone et al., 2019). Therefore, probiotics must be unable to cause hemolysis and gelatin liquefaction in the host. In our study, four strains appear to be safe, as they did not cause erythrocyte lysis in sheep blood and had no gelatinase activity.

Antibacterial susceptibility assays were performed on 12 antimicrobials for phenotypic resistance according to international standards and guidelines. The results confirmed that most strains were susceptible to most antimicrobials and showed strain-dependent characteristics. The results showed consistency with previous studies on a wide range of antimicrobials, therefore no data on resistance genes or cellular localization of resistance genes were available. All strains were resistant to kanamycin, three-quarters were resistant to streptomycin, half were resistant to gentamicin and one-quarter were resistant to ciprofloxacin and rifampicin.

This result is consistent with previous reports that LAB is usually highly resistant to aminoglycosides such as kanamycin, gentamicin and streptomycin. It is considered to be intrinsic in Lactobacillus due to the lack of cytochrome-mediated electron transfer that mediates drug and food uptake (Pradhan et al., 2020).According to previous reports, the intrinsic resistance is chromosomally encoded and poses no risk of horizontal metastasis in non-pathogenic bacteria. In contrast, acquired resistance, caused by the transfer of resistance genes from the environment or from other bacteria, and might be horizontally spread among bacteria (Jeong et al., 2021).

Previous studies showed that 34% of LAB isolated from microbial food and drug additives were resistant to ciprofloxacin (Liu et al., 2009) and 26% of *Lactobacillus* spp. isolated from dairy products were resistant to ciprofloxacin (Erginkaya Z and Tatlı, 2017). *L. acidophilus* and *L. brevis* have been reported to be resistant to ciprofloxacin (Shazali et al., 2014). As Reported by Liu et al. (2009) *L. lactis* strains were all resistant to rifampicin, but he mechanism is not yet clear. Fguiri et al. (2016) showed that *L. plantarum*, *L. pentosus*, *L. brevis*, *L. Lactis* and *Pediococcus pentosaceus* isolated from raw camel milk were resistant to rifampicin. The resistance mechanism of some LAB to ciprofloxacin and rifampicin is still unclear, and we assumed it is a unique characteristic of the strain, which may be related to its origin and evolution.

Finally, comparative analysis of the experimental results showed that FY4 performed best in terms of aggregation, hydrophobicity, antibacterial activities and adherence to Caco-2 cells. FY3 exhibited a high degree of self-aggregation and the strongest antimicrobial activity against *E. coli* and *Salm. Typhimurium*; and FY4 possessed the most potent antimicrobial activity toward three pathogenic bacteria. Previous experimental studies have shown that a mixture of LAB to prevent and treat diarrhea has yielded encouraging results. We speculate that a mixture of FY3 and FY4 may be effective in the prevention and/or treatment of bovine diarrhea. This hypothesis needs to be validated by additional experiments, such as *in vivo* safety and efficacy evaluation to support its practical application.

Toxicity is a complex phenomenon and *in vitro* safety assays may involve false negatives, as virulence properties may be inactive under the specific conditions of the assay. Genome sequencing is currently an approach to assess the safety risk of non-expression (Papadimitriou et al., 2015). In recent years, the safety evaluation of LAB based on the whole genome sequence has been reported. Due to experimental design and economic budget, multi-genomic studies have to be carried out later. Since some toxicities require active interaction with the host to be triggered, oral toxicity tests were conducted in mice as a basis for assessing the safety of probiotics.

Acute toxicity tests in mice showed that LAB did not cause death. Weight change is an indicator of the health of the mice, too rapid increase or decrease may be a sign of immune dysregulation, organ damage and organism disorder. Compared with controls, there were no significant organ damage and no significant differences in body weight, food intake and organ indices in mice. Therefore, these four strains were considered as non-toxic, safe and effective probiotics.

*In vitro* screening of probiotics was applied in our study, it is a simpler and less costly method. Although some tests seem to be outdated, *In vitro* screening are still used in recent reports and its most important advantage is the ability to screen multiple strains at the same time (Papadimitriou et al., 2015). In recent decades, with the rapid development of bioinformatics and high-throughput technologies, LAB studies have expanded from a few genes of interest to quantify whole-genomes, transcriptomes, proteomes and metabolomes changes. The genetic function, metabolic networks, population inheritance and species divergence of LAB can be analyzed by omics techniques, which can accelerate the selection and transform superior strains and provide a basis for the efficient use of LAB, and hence improve the industrial control of fermentation.

## 5 Conclusions

Four LAB were isolated from yak feces in this study, which were *Latilactobacillus curvatus*, *Weissella cibaria*,*  Limosilactobacillus mucosae*, and *Lactiplantibacillus pentosus*. The probiotic properties of LAB were evaluated by hydrophobicity, acid and bile salt resistance, cell aggregation, compatibility, antimicrobial activity and cell adhesion tests. The safety attributes toward hemolytic activity, gelatinase activity and antimicrobial susceptibility were assessed. The results showed that the four strains had probiotic and safety profiles, which were sensitive to spectral antimicrobials, without hemolytic or gelatinase activity. Among them, FY3 and FY4 exhibited outstanding performances in hydrophobicity, aggregation, antibacterial activity and adhesion to Caco-2 cells. The acute oral toxicity test of LAB had no adverse effect on the weight gain, food utilization rate or organ index of mice. All results indicate that these four LAB strains, especially FY3 and FY4 could be potential probiotics for the prevention and/or treatment of bacterial disease-related diarrhea in yak.

## Data availability statement

The original contributions presented in the study are included in the article/**Supplementary Material**. Further inquiries can be directed to the corresponding authors.

## Ethics statement

The animal study was reviewed and approved by College of Veterinary Medicine, Gansu Agricultural University (License no. GSAU-AEW-2019-0010, **Figure S7**).

## Author contributions

QZh, MW, YP, and QZe conceived and designed the experiments. QZh, MW, XM, ZL, and CJ contributed reagents, materials, and analysis tools. QZh wrote the manuscript. YP, MW, and QZe revised the manuscript. All authors contributed to the article and approved the submitted version.

## Funding

This work was supported by Grants from Gansu Province Outstanding Youth Fund (No. 20JR10RA561), the Innovative Talent Project of Gansu Province (No. 20200624RCC0022), the Fuxi Foundation of Exceptional Talent at Gansu Agricultural University (No. FXRC20130103), and the Control of Infectious Diseases of Cattle and Sheep in Modern Agriculture Herbivorous Animal System of Gansu Province (No. GARS-CS-5).

## Conflict of interest

The authors declare that the research was conducted in the absence of any commercial or financial relationships that could be construed as a potential conflict of interest.

## Publisher’s note

All claims expressed in this article are solely those of the authors and do not necessarily represent those of their affiliated organizations, or those of the publisher, the editors and the reviewers. Any product that may be evaluated in this article, or claim that may be made by its manufacturer, is not guaranteed or endorsed by the publisher.

## Abbreviations

LAB, Lactic acid bacteria; MRS, De Man, Rogosa, and Sharpe; MH, Mueller Hinton; LB, Luria Bertani; CFU, Colony-forming unit; PCR, polymerase chain reaction; PBS, Phosphate buffer saline; DMEM, Dulbecco’s modified Eagle medium; FBS, fetal bovine serum; SPF, specified pathogen-free; CFC, Cell-free culture supernatants; *E. coli*, *Escherichia coli*; *S. aureus*, *Staphylococcus aureus*; *Salm. Typhimurium*, *Salmonella Typhimurium*; *L. curvatus*, *Latilactobacillus curvatus*; *W. cibaria*, *Weissella cibaria*; *L. pentosus*, *Lactiplantibacillus pentosus*; *L. mucosae*, *Limosilactobacillus mucosae*.
