# Supplementary material for: In vitro investigation on lactic acid bacteria isolatedfrom Yak faeces for potential probiotics
Source: Front Cell Infect Microbiol. 2022 Sep 16;12:984537. doi: 10.3389/fcimb.2022.984537 (PMC9523120; doi:10.3389/fcimb.2022.984537)
Supplement: Supplementary file 1 [file DataSheet_1.pdf]

>FY1
GTGCCGTGCGGCGTGCTATACATGCAAGTCGAACGCACTCTCGTTAGATTGAAGAAGCTT
GCTTCTGATTGATAACATTTGAGTGAGTGGCGGACGGGTGAGTAACACGTGGGTAACCTG
CCCTAAAGTGGGGGATAACATTTGGAAACAGATGCTAATACCGCATAAAACCTAGCACCG
CATGGTGCAAGGTTGAAAGATGGTTTCGGCTATCACTTTAGGATGGACCCGCGGTGCATT
AGTTAGTTGGTGAGGTAAAGGCTCACCAAGACCGTGATGCATAGCCGACCTGAGAGGGTA
ATCGGCCACACTGGGACTGAGACACGGCCCAGACTCCTACGGGAGGCAGCAGTAGGGAAT
CTTCCACAATGGACGAAAGTCTGATGGAGCAACGCCGCGTGAGTGAAGAAGGTTTTCGGA
TCGTAAAACTCTGTTGTTGGAGAAGAACGTATTTGATAGTAACTGATCAGGTAGTGACGG
TATCCAACCAGAAAGCCACGGCTAACTACGTGCCAGCAGCCGCGGTAATACGTAGGTGGC
AAGCGTTGTCCGGATTTATTGGGCGTAAAGCGAGCGCAGGCGGTTTCTTAAGTCTGATGT
GAAAGCCTTCGGCTCAACCGAAGAAGTGCATCGGAAACTGGGAAACTTGAGTGCAGAAGA
GGACAGTGGAACTCCATGTGTAGCGGTGAAATGCGTAGATATATGGAAGAACACCAGTGG
CGAAGGCGGCTGTCTGGTCTGTAACTGACGCTGAGGCTCGAAAGCATGGGTAGCAAACAG
GATTAGATACCCTGGTAGTCCATGCCGTAAACGATGAGTGCTAGGTGTTGGAGGGTTTCC
GCCCTTCAGTGCCGCAGCTAACGCATTAAGCACTCCGCCTGGGGAGTACGACCGCAAGGT
TGAAACTCAAAGGAATTGACGGGGGCCCGCACAAGCGGTGGAGCATGTGGTTTAATTCGA
AGCAACGCGAAGAACCTTACCAGGTCTTGACATCCTTTGACCACTCTAGAGATAGAGCTT
TCCCTTCGGGGACAAAGTGACAGGTGGTGCATGGTTGTCGTCAGCTCGTGTCGTGAGATG
TTGGGTTAAGTCCCGCAACGAGCGCAACCCTTATTACTAGTTGCCAGCATTTAGTTGGGC
ACTCTAGTGAGACTGCCGGTGACAAACCGGAGGAAGGTGGGGACGACGTCAAATCATCAT
GCCCCTTATGACCTGGGCTACACACGTGCTACAATGGATGGTACAACGAGTCGCGAGACC
GCGAGGTTTAGCTAATCTCTTAAAACCATTCTCAGTTCGGATTGTAGGCTGCAACTCGCC
TACATGAAGCCGGAATCGCTAGTAATCGCGGATCAGCATGCCGCGGTGAATACGTTCCCG
GGCCTTGTACACACCGCCCGTCACACCATGAGAGTTTGTAACACCCAAAGCCGGTGAGGT
AACCTTCGGGAGCCAGCCGTCTAAGGTGACAGATGAG

>FY2
CTATACATGCAGTCGAACGCTTTGTGGTTCAACTGATTTGAAGAGCTTGCTCAGATATGA
CGATGGACATTGCAAAGAGTGGCGAACGGGTGAGTAACACGTGGGAAACCTACCTCTTAG
CAGGGGATAACATTTGGAAACAGATGCTAATACCGTATAACAATAGCAACCGCATGGTTG
CTACTTAAAAGATGGTTCTGCTATCACTAAGAGATGGTCCCGCGGTGCATTAGTTAGTTG
GTGAGGTAATGGCTCACCAAGACGATGATGCATAGCCGAGTTGAGAGACTGATCGGCCAC
AATGGGACTGAGACACGGCCCATACTCCTACGGGAGGCAGCAGTAGGGAATCTTCCACAA
TGGGCGAAAGCCTGATGGAGCAACGCCGCGTGTGTGATGAAGGGTTTCGGCTCGTAAAAC
ACTGTTGTAAGAGAAGAATGACATTGAGAGTAACTGTTCAATGTGTGACGGTATCTTACC
AGAAAGGAACGGCTAAATACGTGCCAGCAGCCGCGGTAATACGTATGTTCCAAGCGTTAT
CCGGATTTATTGGGCGTAAAGCGAGCGCAGACGGTTATTTAAGTCTGAAGTGAAAGCCCT
CAGCTCAACTGAGGAATTGCTTTGGAAACTGGATGACTTGAGTGCAGTAGAGGAAAGTGG
AACTCCATGTGTAGCGGTGAAATGCGTAGATATATGGAAGAACACCAGTGGCGAAGGCGG
CTTTCTGGACTGTAACTGACGTTGAGGCTCGAAAGTGTGGGTAGCAAACAGGATTAGATA
CCCTGGTAGTCCACACCGTAAACGATGAGTGCTAGGTGTTTGAGGGTTTCCGCCCTTAAG
TGCCGCAGCTAACGCATTAAGCACTCCGCCTGGGGAGTACGACCGCAAGGTTGAAACTCA
AAGGAATTGACGGGGACCCGCACAAGCGGTGGAGCATGTGGTTTAATTCGAAGCAACGCG

AAGAACCTTACCAGGTCTTGACATCCCTTGACAACTCCAGAGATGGAGCGTTCCCTTCGG
GGACAAGGTGACAGGTGGTGCATGGTTGTCGTCAGCTCGTGTCGTGAGATGTTGGGTTAA
GTCCCGCAACGAGCGCAACCCTTATTACTAGTTGCCAGCATTCAGTTGGGCACTCTAGTG
AGACTGCCGGTGACAAACCGGAGGAAGGTGGGGATGACGTCAAATCATCATGCCCCTTAT
GACCTGGGCTACACACGTGCTACAATGGCGTATACAACGAGTTGCCAACCCGCGAGGGTG
AGCTAATCTCTTAAAGTACGTCTCAGTTCGGATTGTAGGCTGCAACTCGCCTACATGAAG
TCGGAATCGCTAGTAATCGCGGATCAGCACGCCGCGGTGAATACGTTCCCGGGTCTTGTA
CACACCGCCCGTCACACCATGAGAGTTTGTAACACCCAAAGCCGGTGGGGTAACCTTCGG
GAGCCAGCCGTCTAAGG

>FY3
GGGCCGGGCGCGTGCTATACATGCAAGTCGAACGCGTTGGCCCAACTGATTGAACGTGCT
TGCACGGACTTGACGTTGGTTTACCAGCGAGTGGCGGACGGGTGAGTAACACGTAGGTAA
CCTGCCCCAAAGCGGGGGATAACATTTGGAAACAGATGCTAATACCGCATAACAATTTGA
ATCGCATGATTCAAATTTAAAAGATGGCTTCGGCTATCACTTTGGGATGGACCTGCGGCG
CATTAGCTTGTTGGTAGGGTAACGGCCTACCAAGGCTGTGATGCGTAGCCGAGTTGAGAG
ACTGATCGGCCACAATGGAACTGAGACACGGTCCATACTCCTACGGGAGGCAGCAGTAGG
GAATCTTCCACAATGGGCGCAAGCCTGATGGAGCAACACCGCGTGAGTGAAGAAGGGTTT
CGGCTCGTAAAGCTCTGTTGTTAGAGAAGAACGTGCGTGAGAGCAACTGTTCACGCAGTG
ACGGTATCTAACCAGAAAGTCACGGCTAACTACGTGCCAGCAGCCGCGGTAATACGTAGG
TGGCAAGCGTTATCCGGATTTATTGGGCGTAAAGCGAGCGCAGGCGGTTTGATAAGTCTG
ATGTGAAAGCCTTTGGCTTAACCAAAGAAGTGCATCGGAAACTGTCAGACTTGAGTGCAG
AAGAGGACAGTGGAACTCCATGTGTAGCGGTGGAATGCGTAGATATATGGAAGAACACCA
GTGGCGAAGGCGGCTGTCTGGTCTGCAACTGACGCTGAGGCTCGAAAGCATGGGTAGCGA
ACAGGATTAGATACCCTGGTAGTCCATGCCGTAAACGATGAGTGCTAGGTGTTGGAGGGT
TTCCGCCCTTCAGTGCCGCAGCTAACGCATTAAGCACTCCGCCTGGGGAGTACGACCGCA
AGGTTGAAACTCAAAGGAATTGACGGGGGCCCGCACAAGCGGTGGAGCATGTGGTTTAAT
TCGAAGCTACGCGAAGAACCTTACCAGGTCTTGACATCTTGCGCCAACCCTAGAGATAGG
GCGTTTCCTTCGGGAACGCAATGACAGGTGGTGCATGGTCGTCGTCAGCTCGTGTCGTGA
GATGTTGGGTTAAGTCCCGCAACGAGCGCAACCCTTGTTACTAGTTGCCAGCATTCAGTT
GGGCACTCTAGTGAGACTGCCGGTGACAAACCGGAGGAAGGTGGGGACGACGTCAGATCA
TCATGCCCCTTATGACCTGGGCTACACACGTGCTACAATGGACGGTACAACGAGTCGCGA
ACTCGCGAGGGCAAGCTAATCTCTTAAAACCGTTCTCAGTTCGGACTGCAGGCTGCAACT
CGCCTGCACGAAGTCGGAATCGCTAGTAATCGCGGATCAGCATGCCGCGGTGAATACGTT
CCCGGGCCTTGTACACACCGCCCGTCACACCATGAGAGTTTGCAACACCCAAAGTCGGTG
GGGTAACCCTTCGGGGAGCTAGCCGCCTAAGGTGTAGAAATGT

>FY4
GGCAGTCGGGGTGCTATACATGCAGTCGAACGAACTCTGGTATTGATTGGTGCTTGCATCATGA
TTTACATTTGAGTGAGTGGCGAACTGGTGAGTAACACGTGGGAAACCTGCCCAGAAGCGGGGG
ATAACACCTGGAAACAGATGCTAATACCGCATAACAACTTGGACCGCATGGTCCGAGTTTGAAA
GATGGCTTCGGCTATCACTTTTGGATGGTCCCGCGGCGTATTAGCTAGATGGTGGGGTAACGGC
TCACCATGGCAATGATACGTAGCCGACCTGAGAGGGTAATCGGCCACATTGGGACTGAGACAC
GGCCCAAACTCCTACGGGAGGCAGCAGTAGGGAATCTTCCACAATGGACGAAAGTCTGATGGA

GCAACGCCGCGTGAGTGAAGAAGGGTTTCGGCTCGTAAAACTCTGTTGTTAAAGAAGAACATAT
CTGAGAGTAACTGTTCAGGTATTGACGGTATTTAACCAGAAAGCCACGGCTAACTACGTGCCAG
CAGCCGCGGTAATACGTAGGTGGCAAGCGTTGTCCGGATTTATTGGGCGTAAAGCGAGCGCAG
GCGGTTTTTTAAGTCTGATGTGAAAGCCTTCGGCTCAACCGAAGAAGTGCATCGGAAACTGGGA
AACTTGAGTGCAGAAGAGGACAGTGGAACTCCATGTGTAGCGGTGAAATGCGTAGATATATGG
AAGAACACCAGTGGCGAAGGCGGCTGTCTGGTCTGTAACTGACGCTGAGGCTCGAAAGTATGG
GTAGCAAACAGGATTAGATACCCTGGTAGTCCATACCGTAAACGATGAATGCTAAGTGTTGGAG
GGTTTCCGCCCTTCAGTGCTGCAGCTAACGCATTAAGCATTCCGCCTGGGGAGTACGGCCGCAA
GGCTGAAACTCAAAGGAATTGACGGGGGCCCGCACAAGCGGTGGAGCATGTGGTTTAATTCGA
AGCTACGCGAAGAACCTTACCAGGTCTTGACATACTATGCAAATCTAAGAGATTAGACGTTCCC
TTCGGGGACATGGATACAGGTGGTGCATGGTTGTCGTCAGCTCGTGTCGTGAGATGTTGGGTTA
AGTCCCGCAACGAGCGCAACCCTTATTATCAGTTGCCAGCATTAAGTTGGGCACTCTGGTGAGA
CTGCCGGTGACAAACCGGAGGAAGGTGGGGATGACGTCAAATCATCATGCCCCTTATGACCTG
GGCTACACACGTGCTACAATGGATGGTACAACGAGTTGCGAACTCGCGAGAGTAAGCTAATCT
CTTAAAGCCATTCTCAGTTCGGATTGTAGGCTGCAACTCGCCTACATGAAGTCGGAATCGCTAG
TAATCGCGGATCAGCATGCCGCGGTGAATACGTTCCCGGGCCTTGTACACACCGCCCGTCACA
CCATGAGAGTTTGTAACACCCAAAGTCGGTGGGGTAACCTTTTAGGAACCAGCCGCTAAGGTG
ACAGGTTGGG
