# Supplementary material for: In vitro investigation on lactic acid bacteria isolatedfrom Yak faeces for potential probiotics
Source: Front Cell Infect Microbiol. 2022 Sep 16;12:984537. doi: 10.3389/fcimb.2022.984537 (PMC9523120; doi:10.3389/fcimb.2022.984537)
Supplement: Supplementary file 2 [file DataSheet_2.pdf]

Supplement legend

Supplement 1
Figure 1S The location of sample collection and sample container. A: The location of sample collection B: The sample container

Supplement 2
Figure 2S The characteristic morphologies of colony and cell of isolated LAB.
A: FY1; B: FY2; C: FY3; D: FY4

Supplement 3
Figure 3S Amplification results of 16S rRNA gene from four isolates.
Lane M: molecular weight marker 2kb; Lane 1: FY1; Lane 2: FY2; Lane 3: FY3; Lane 4: FY4

Supplement 4
Figure 4S Results of compatibility test of LAB A: FY1; B: FY2; C: FY3; D: FY4; 1: CFC of FY1; 2: CFC of FY2; 3: CFC of FY3; 4: CFC of FY4.

Supplement 5
Figure 5S The hemolytic activity results of LAB isolates.
A: FY1; B: FY2; C: FY3; D: FY4; E: *S. aureus*

Supplement 6
Figure 6S Results of gelatinase test of LAB

Supplement 7
Figure 7S Affidavit of approval of animal ethical and welfare

Supplement 8
Data Sheet 1 The sequences of 16S rRNA gene.

Supplement 9
Data Sheet 2 Supplement legend
