# Supplementary figures and images for: In vitro investigation on lactic acid bacteria isolatedfrom Yak faeces for potential probiotics
Source: Front Cell Infect Microbiol. 2022 Sep 16;12:984537. doi: 10.3389/fcimb.2022.984537 (PMC9523120; doi:10.3389/fcimb.2022.984537)

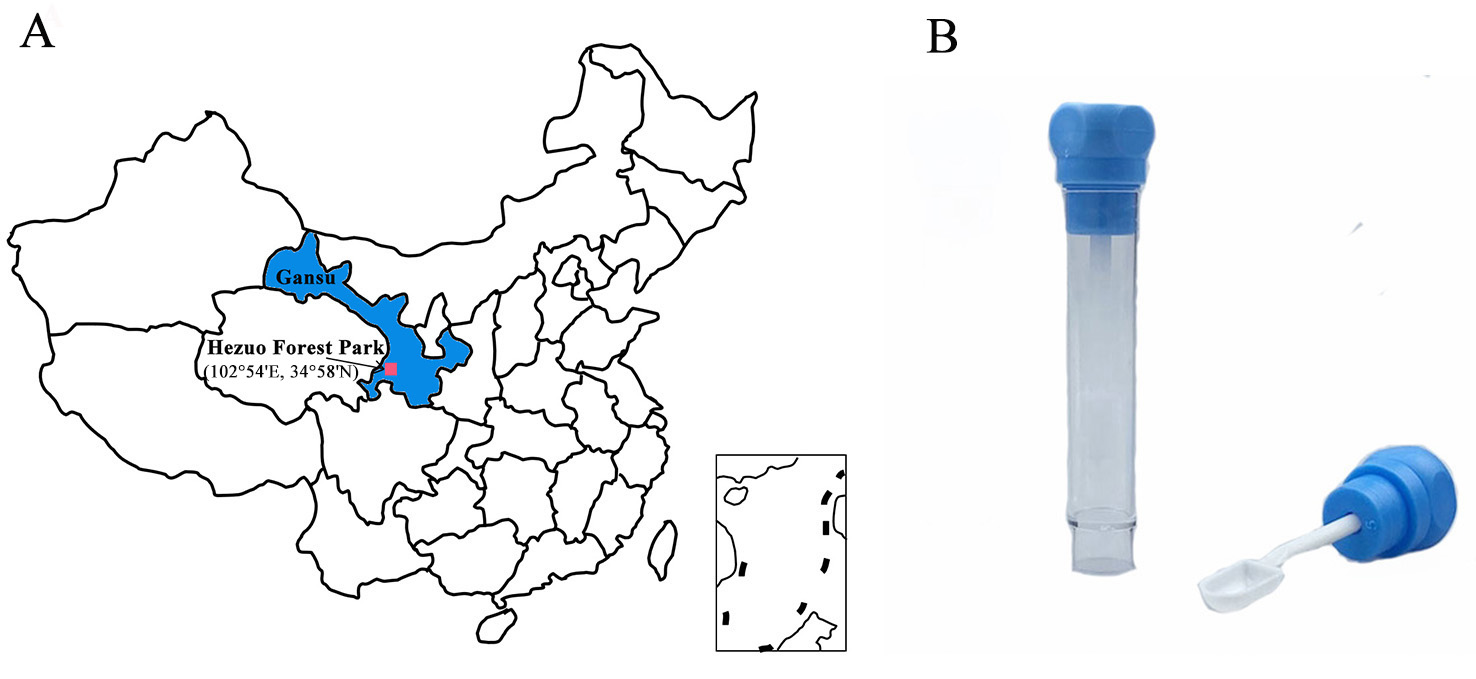

Supplement: Supplementary file 3 [file Image_1.jpeg]

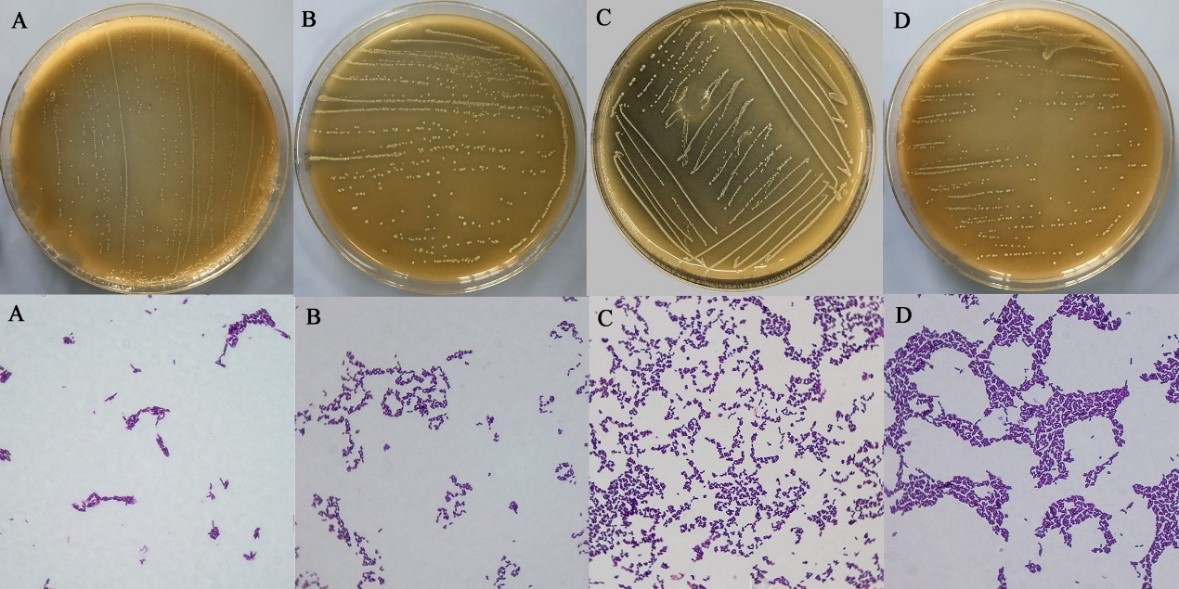

Supplement: Supplementary file 4 [file Image_2.jpeg]

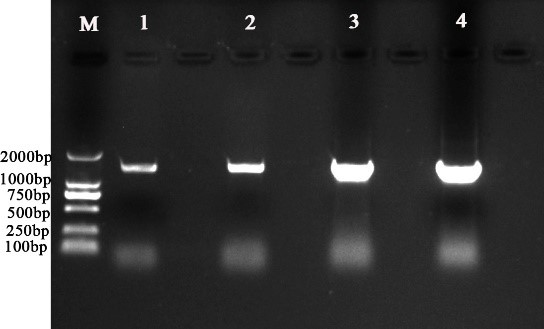

Supplement: Supplementary file 5 [file Image_3.jpeg]

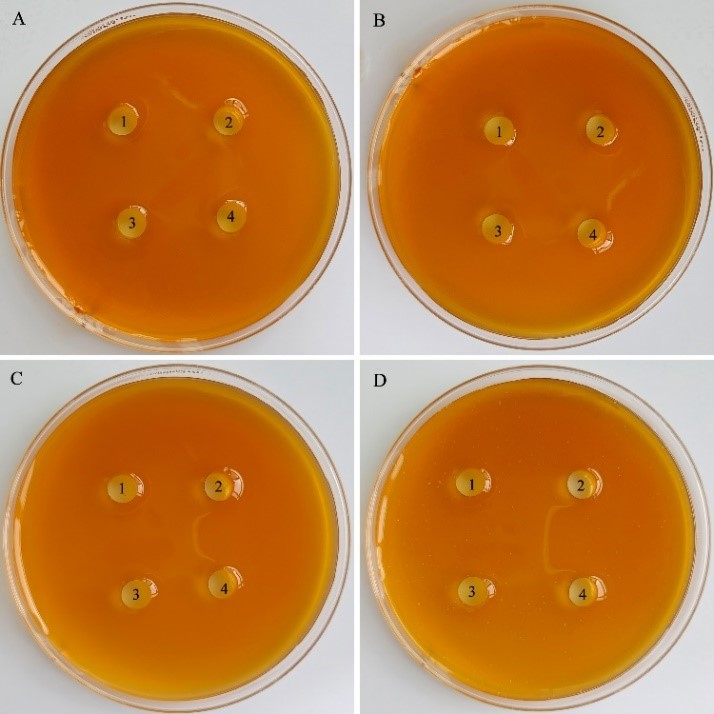

Supplement: Supplementary file 6 [file Image_4.jpeg]

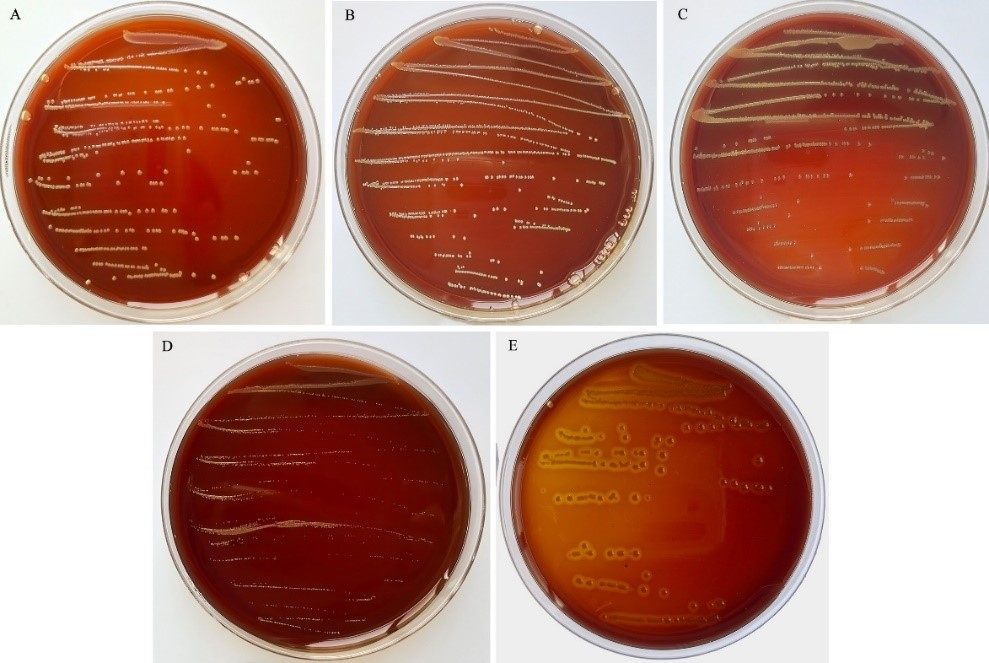

Supplement: Supplementary file 7 [file Image_5.jpeg]

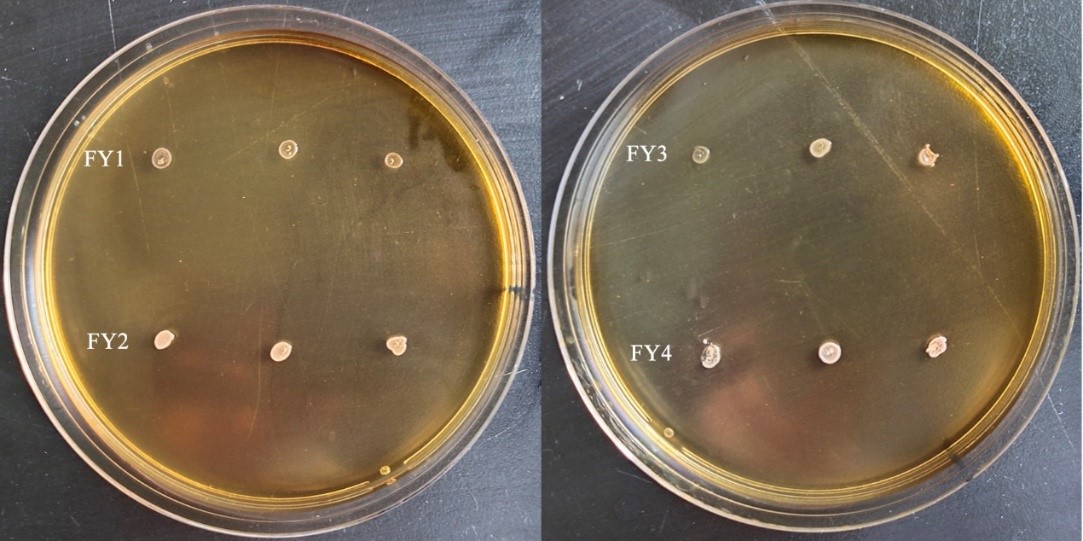

Supplement: Supplementary file 8 [file Image_6.jpeg]

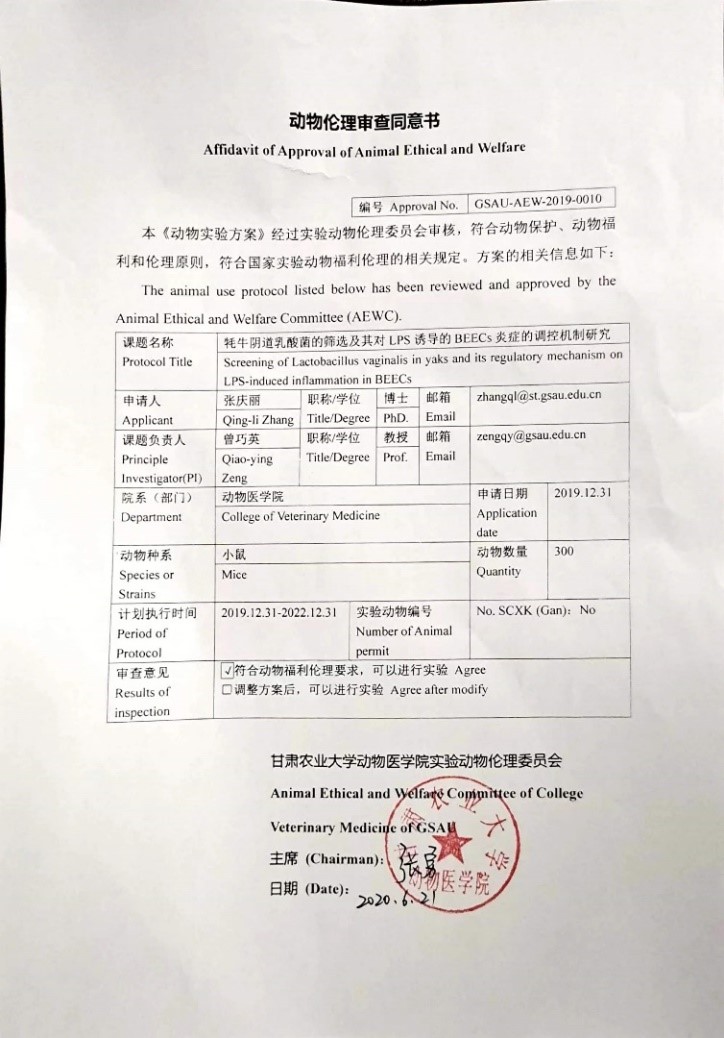

Supplement: Supplementary file 9 [file Image_7.jpeg]
